# Is it possible to make ‘living’ guidelines? An evaluation of the Australian Living Stroke Guidelines

**DOI:** 10.1186/s12913-024-10795-6

**Published:** 2024-04-03

**Authors:** Louise Wiles, Peter D Hibbert, Yvonne Zurynski, Carolynn L. Smith, Gaston Arnolda, Louise A. Ellis, Rebecca Lake, Brona Nic Giolla Easpaig, Charlotte Molloy, Sandy Middleton, Jeffrey Braithwaite, Kelvin Hill, Tari Turner

**Affiliations:** 1 Australian Institute of Health Innovation, Macquarie University, Sydney, NSW Australia; 2 Innovation, Implementation & Clinical Translation (IIMPACT) in Health, University of South Australia, Adelaide, Australia; 3 South Australian Health and Medical Research Institute, Adelaide, Australia; 4 NHMRC Partnership Centre for Health System Sustainability, Sydney, Australia; 5 Nursing Research Institute, St Vincent’s Health Network Sydney and Australian Catholic University, Sydney, Australia; 6 The Stroke Foundation, Sydney, Australia; 7 School of Population Health and Preventive Medicine, Monash University, Melbourne, Australia

**Keywords:** Living guidelines, Clinical guidelines, Guideline adherence, Evidence based healthcare management

## Abstract

**Background:**

Keeping best practice guidelines up-to-date with rapidly emerging research evidence is challenging. ‘Living guidelines’ approaches enable continual incorporation of new research, assisting healthcare professionals to apply the latest evidence to their clinical practice. However, information about how living guidelines are developed, maintained and applied is limited. The Stroke Foundation in Australia was one of the first organisations to apply living guideline development methods for their Living Stroke Guidelines (LSGs), presenting a unique opportunity to evaluate the process and impact of this novel approach.

**Methods:**

A mixed-methods study was conducted to understand the experience of LSGs developers and end-users. We used thematic analysis of one-on-one semi-structured interview and online survey data to determine the feasibility, acceptability, and facilitators and barriers of the LSGs. Website analytics data were also reviewed to understand usage.

**Results:**

Overall, the living guidelines approach was both feasible and acceptable to developers and users. Facilitators to use included collaboration with multidisciplinary clinicians and stroke survivors or carers. Increased workload for developers, workload unpredictability, and limited information sharing, and interoperability of technological platforms were identified as barriers. Users indicated increased trust in the LSGs (69%), likelihood of following the LSGs (66%), and frequency of access (58%), compared with previous static versions. Web analytics data showed individual access by 16,517 users in 2016 rising to 53,154 users in 2020, a threefold increase. There was also a fourfold increase in unique LSG pageviews from 2016 to 2020.

**Conclusions:**

This study, the first evaluation of living guidelines, demonstrates that this approach to stroke guideline development is feasible and acceptable, that these approaches may add value to developers and users, and may increase guideline use. Future evaluations should be embedded along with guideline implementation to capture data prospectively.

**Supplementary Information:**

The online version contains supplementary material available at 10.1186/s12913-024-10795-6.

## Background

The COVID-19 pandemic brought the problem with traditional evidence-based guideline development and dissemination to the forefront, as policy-makers and health professionals struggled to keep up with the latest research [1]. The methods for developing rigorous evidence-based guidelines [2, 3] are resource intensive and time-consuming, leading to delays in publishing and updating guidelines, thereby delaying their availability to assist in clinical decision-making and, with implementation support, the potential for translation of research evidence into improved healthcare practices and patient outcomes [4]. Worse, many guidelines do not incorporate the latest evidence and are out-of-date before they are even published [5]. Living guidelines methods, such as those employed by local jurisdictions [6] and internationally [7–14], aim to overcome such delays, enabling the rigour of systematic review processes to be maintained while continually identifying and incorporating new research [15]. Living guidelines do not represent a new approach, but rather an optimization of standard guideline development methods [15], with two important modifications: frequent surveillance for (often monthly review of) new studies, and updating of individual recommendations (instead of the whole guideline) as relevant new evidence becomes available [15, 16]. 

In 2017, the Stroke Foundation in Australia (Stroke Foundation) partnered with Cochrane Australia to become one of the first organisations in the world to transition to living guideline development methods [17, 18]. The 2017 Stroke Foundation guidelines included more than 300 recommendations addressing over 80 clinical topics [19]. This transition provided a unique opportunity to evaluate the development of living guidelines and their impact. Supported by the Australian Government in 2018, a pilot study was launched to test the creation of a near real-time, closed-loop evidence system in which global evidence and local data are continually integrated with clinical expertise.

**Fig. 1:**
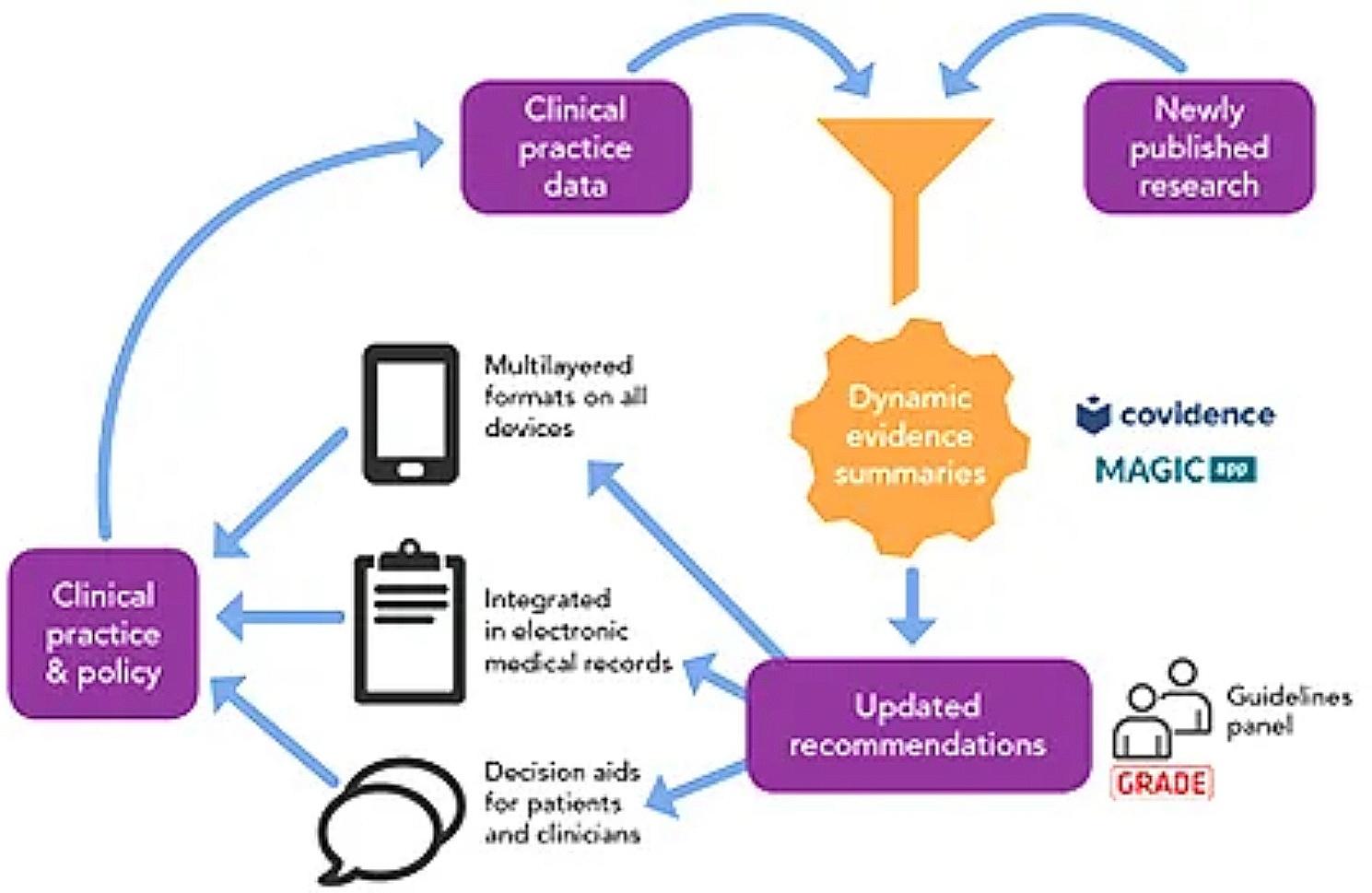
The Stroke Foundation’s Living Stroke Guideline development methods

Maintenance of the Living Stroke Guidelines (LSGs) involved monthly surveillance of the literature for new systematic reviews and randomised controlled studies [16]. Relevant studies underwent data extraction followed by preparation of updated evidence-to-decision frameworks which were then used to inform updates, or to develop new recommendations. Small writing groups (as used in standard guideline development approaches), comprised of clinical experts and people with lived stroke experience, reviewed and reached consensus on changes, which were vetted by a multidisciplinary Guidelines Steering Group. Changes were developed and published using the online collaborative tool MAGICapp (also used in standard digital clinical practice guidelines) [20], which assists authors with writing and publishing highly structured guidelines and evidence summaries, and enables users to access multilayered information, including the strength of evidence for each recommendation [20]. Dissemination occurs via email alerts for registered users and social media posts [16]. Figure 1 describes the process.

## Aim

In this study we aimed to:

1. Explore the impact of the LSGs approach on workload and efficiency of guideline production *(feasibility)*;
2. Identify facilitators and barriers for living guidelines development and production and how these can be leveraged or overcome to inform future processes; and
3. Investigate how the LSGs approach influenced *acceptability* of the guideline recommendations among end-users.

## Methods

In October 2020, we conducted a mixed-methods (realist paradigm) evaluation [21, 22], including sequential quantitative surveys and qualitative interviews to understand the experience of stakeholders contributing to, or impacted by, living guideline production and implementation (additional files 1 and 2). Google Analytics data were used to examine access trends over five years. Macquarie University Human Research Ethics Committee granted ethics approval (ID: 7918).

### Participants

The evaluation included two groups of participants:

- Guideline developers—The Stroke Foundation guideline development group, including the project team, executive, guideline content and methodology experts, people with lived experience of stroke;
- Guideline users—stakeholder group members and end-users, including clinicians.

Participants were invited through a link to an online survey sent through existing Stroke Foundation email lists. Participation was voluntary, and the survey included a participant information statement. Choosing to proceed with the survey constituted informed consent. At the end of the survey, participants could choose to provide their contact details if they were willing to undertake a follow-up interview.

### Data collection and analysis

#### Surveys

Survey data were collected using the REDCap (version REDCap10.0.6) online survey tool in October 2020. Surveys included demographic details, closed-ended and open-ended questions about guideline developers’ experiences with the LSGs production or guideline users’ opinions about acceptability, perceived utility and reliability, and likelihood of accessing LSGs.

De-identified data from the surveys were summarised using descriptive statistics. Thematic analysis was performed on the answers to open-ended questions to identify common themes.

#### Interviews

Interview data collection and analyses were conducted and are described according to the Consolidated Criteria for Reporting Qualitative Studies (COREQ) [23] (additional file 3). Guideline developers and users were invited to participate in interviews through the Stroke Foundation email lists and via a link in the survey. Interview participants returned a signed consent form by email after reading the participant information statement (which included researchers’ role and qualification details). They were then contacted by the interviewer to schedule an interview time, respond to any questions and establish a relationship prior to data collection.

Semi-structured interview schedules (additional files 4 and 5) were developed in partnership with the Stroke Foundation, reviewed by two stroke experts and pilot-tested with three study participants. No changes were made following the pilot study. These data are included in the study. The schedules included collection of demographic information, and a range of closed- and open-ended questions. The interviewers (RL, LW) were female, experienced in qualitative methodology, employed Research Assistant (RL) and Research Fellow (LW, and had background qualifications in Social Sciences (Bachelor degree, RL) and Health Sciences/Physiotherapy (PhD/Bachelor with Honours degrees, LW). Prompts were used to probe for more detailed responses as required.

One-on-one interviews were conducted via telephone or web-based platform (Zoom, version 5.13.3) and explored participants’ experiences of the guideline production (developers) or guideline dissemination and translation into practice (users); perceived facilitators and barriers of the LSGs approach; and opportunities to improve the current production model and translation approach. Interviews were audio-recorded (with field notes taken), transcribed verbatim and de-identified. No repeat interviews were carried out. Interview transcripts and summaries of the analysis were sent to participants to ensure they were satisfied with interpretations of their data, with no suggested amendments received.

An inductive thematic analysis of the transcribed interview data was undertaken using a team-based approach. NVivo (version 12) was used to code the data. Initially, three researchers (LKW, RL, BNGE) independently open-coded 10% of the interview data across both participant groups to identify distinct concepts and themes. Comparison and discussion among the researchers facilitated the development of an analytical framework, which was subsequently applied in the analysis of the remaining data. Cross-checking was used to independently verify data saturation, and the validity and reliability of the coding strategy for the remaining data, alongside ongoing team discussion of the emerging analysis.

#### Integrated analysis

Following individual analysis, the website analytics (quantitative), survey (quantitative and qualitative) and interview (qualitative) data were integrated according to a triangulation protocol [24]. The original data, themes and summaries were organised into a ‘matrix’ and examined by LW, PH, KH; ‘meta-themes’ that cut across all data sources were identified and used to craft key findings through discussion and consensus [24]. 

## Results

### Participants

#### Guideline developers Survey

Of the 146 guideline developers invited, 50 (34%) completed at least one question, and 39 (27%) completed the entire survey. The 50 respondents had 57 roles; 21 (37%) were guideline content development group members, 14 (25%) people with lived experience of stroke, 10 (18%) on the LSG project team, and nine (16%) on the guideline advisory committee. Seven people had two roles. Participants reported being involved in guideline production for between 1 and 20 years (median 2.5 years).

#### Guideline users Survey

From the Stroke Foundation’s email circulation list of 18,240 members, 178 guideline users (1.0%) completed at least one survey question. Participants had a mean age of 45 years (min: 21, max: 75, median: 47), and averaged 19 and 11 years professional and stroke-specific experience respectively. Of 156 respondents who completed the entire survey, 86% (*n* = 134) were aware of the LSGs with 74% (*n* = 115) having used them. Of the 115 who had previously used the guidelines, the most common reasons given for accessing them were: to inform clinical practice both in general/professional development (*n* = 88, 77%), for a specific situation/patient (*n* = 76, 66%), or to guide clinical practice improvement or professional development within their team (*n* = 77, 67%). 77%, 103 of the 133 who responded to the question, indicated that they had used the previous static stroke guidelines, with an additional 18% (*n* = 24) aware of them but not having used them.

#### Interviews

Interviews (*n* = 29; 14 developers, 15 users) were conducted between November 2020 and April 2021 and ran for a mean duration of 30 min and 14 s (median: 28 min, 11 s; range 11 min, 36 s– 52 min, 48 s). No participants declined to participate or dropped out. The guideline developers were located in four (of eight) Australian states and territories (New South Wales: 6, Victoria: 4, Queensland: 3, Western Australia: 1), and included a mix of health professionals (*n* = 7; including four allied health practitioners, of whom, three were also researchers) and people with lived experience (*n* = 5 stroke survivors, *n* = 2 carers). There was an even distribution of participants with and without prior experience in evidence synthesis and guideline production. Guideline developers’ self-reported health professional and lived experience profiles are presented in Table 1.

**Table 1:** Self-reported profiles of interviewed guideline developers

Self-reported profile	N (%)
*Health professionals*	
Speech Pathologist / Researcher^#^	2 (14%)
Speech Pathologist^#^	1 (7%)
Neurologist	1 (7%)
Physiotherapist / Researcher^#^	1 (7%)
Paramedic	1 (7%)
Project Coordinator (Speech Pathologist)	1 (7%)
*Lived experience*	
Stroke survivor^	5 (37%)
Carer of stroke survivor*	2 (14%)
TOTAL	14 (100%)

^#^*n* = 4 allied health professionals

^ *n* = 4 stroke survivors (mean current age: 57.8 years, range 48–70 years; mean age of stroke onset: 44.3 years, range 18–64 years; impairments: hemiparesis, hand weakness, aphasia, reduced mobility, fatigue, balance and visual impairments); *n* = 1 stroke survivor (no details provided)

* *n* = 1 carer of stroke survivor (working age) 6 years prior, *n* = 1 carer of stroke survivor (daughter) 7 years prior

The guideline users included participants from six Australian states (Queensland: 6, New South Wales: 3, Victoria: 3; one each from South Australia, Tasmania, and Western Australia). Twelve of these were experienced medical educators and/or had been involved in developing quality improvement programs or evidence-syntheses. Guideline users’ self-reported professional roles are presented in Table 2.

**Table 2:** Self-reported professional roles of interviewed guideline users

Self-reported position titles	N (%)
Stroke Clinical Nurse Consultant	5 (33)
Stroke Coordinator (hospital, district, state-wide network)	3 (20)
Medical Staff Specialist / Director (Neurology Department)	2 (13)
Senior Allied Health Professional (Stroke/Neurology Unit) ^	2 (13)
Manager / Clinical Lead (non-stroke specific) *	2 (13)
Stroke Case Manager	1 (7)
TOTAL	15 (100%)

^ *n* = 1 Occupational Therapist, *n* = 1 Speech Pathologist

* *n* = 1 Manager Strategic Clinical Change (Rural Support Service), *n* = 1 Project Manager/Lead (Clinical Telehealth)

**Fig. 2:**
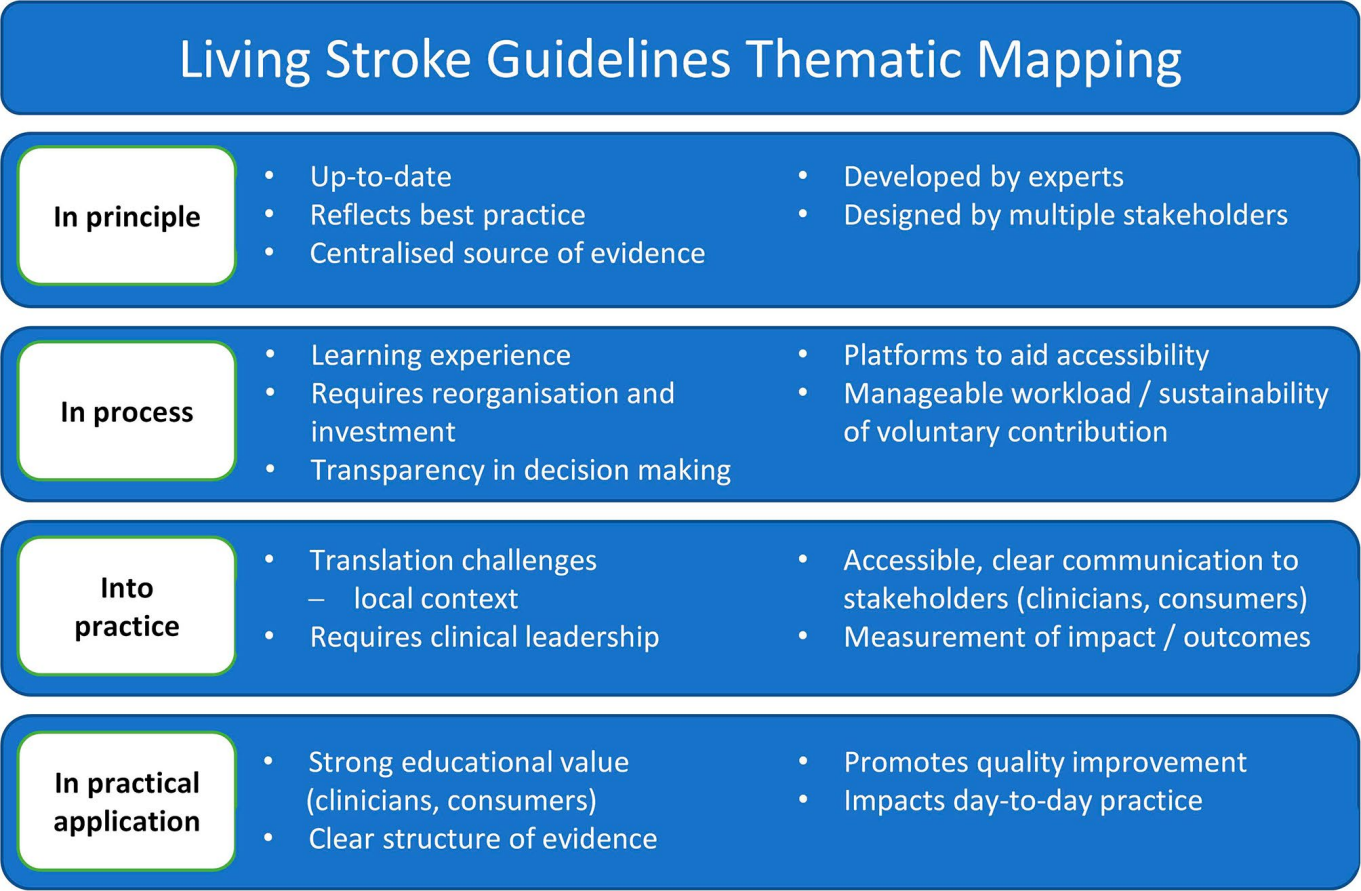
Thematic mapping of interview data

Four themes were crafted from the interview data analysis, which captured participants’ experiences within and around the ongoing LSG cycle (Fig. 2). The themes also included identified factors that form barriers and facilitators to success of the development and use of LSGs from developers’ and users’ perspectives, respectively (Table 3).

### Key findings

The findings were congruent between the two survey groups and across the themes identified from interviews with guideline developers and users. Therefore, we provide a synthesis of findings from all three data sources (website analytics, surveys, and interviews).

#### Acceptability and impact of living guideline approach

Guideline users surveyed were more likely to follow the recommendations (66% of 114 respondents), intended to use them more frequently (58% of 115), were more likely to access the LSGs (63% of 116), and had increased trust in the LSGs over previous static versions (69% of 116).

*“Well look, I think they’re working well. As I said, because you do know when you log on that you are getting the most up-to-date information, and you are getting what is the expected standard in Australia.”* (*Guideline user, Interview Participant 12*).

**Fig. 3:**
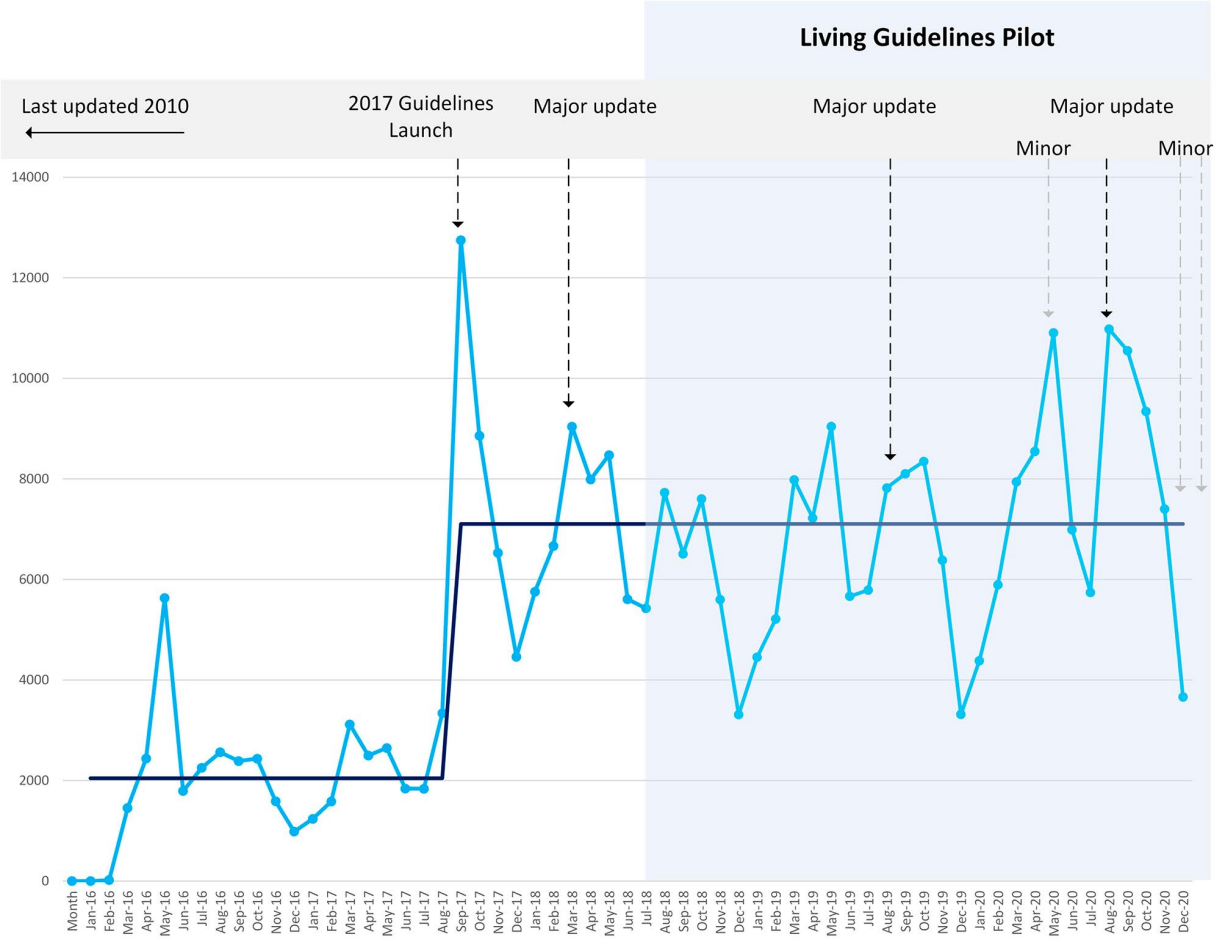
Unique pageviews of the guidelines 2016–2020. Note: *Darker line is the median of unique monthly pageviews prior to and after the release of the guidelines in 2017*

Google Analytics data showed that the number of individual users accessing the guidelines increased by 222% from 16,517 in 2016 to 53,154 in 2020. Unique pageviews of the guidelines increased four-fold after their release in September 2017 (from 7,013 over June-August to 28,131 over September-November), and by 292% (from 23,535 in 2016 to 92,327 in 2020) following the 2017 update after commencement of the LSGs pilot in 2018 (Fig. 3).

#### Feasibility of the living guideline approach and processes

The survey and interview data show that convening and coordinating skills are vital to the success of living guidelines programs, which was a strength of the Stroke Foundation team.

*“By far, the success of these guidelines is due to the strong and committed team leading this project… They are on top of all processes and communication around the guideline development and are doing a stellar job in coordinating the expert input of extraordinarily busy clinician-researchers.”* (*Guideline developer, Survey Participant 9*).

The majority (83%of 48 respondents) of guideline developers agreed that the way different groups were involved in the production of the living guidelines worked well. A frequently highlighted strength of the process was the broad range of input, including multidisciplinary clinical representation and strong stroke survivor/carer involvement, especially through face-to-face or on-line meetings in the initial phases of development.

*“I think what works well is the balancing of medical professional advice with the lived experience of consumers as this does not always mirror the medical advice.”* (*Guideline developer, Survey Participant 32*).

Almost 90% of guideline developers (*n* = 43/46) reported that the processes underpinning the production of the LSGs work well. However, one noted that arriving at the operational details of those processes was challenging:

*“…when the rubber hits the road and you have to make really practical decisions about how you actually do this/ that, that’s been exceptionally challenging to get the right mix of rigour and sustainability and workload across the people who are doing this.”* (*Guideline developer, Interview Participant 5*).

Over half of guideline developers (*n* = 23/41, 54%) stated that the LSGs process increased their workload. About two-thirds (n=15/23, 65%) of those reporting an increase in their workload attributed this to updates being done more regularly (*n* = 7), being new to the process (*n* = 3), being unfamiliar with the process (*n* = 3), and having greater involvement in the process (*n* = 2). Four developers who said their workload decreased suggested this was because of the incremental nature of living guideline development (i.e., whilst updates are more frequent less work is required for each update).

Guideline developers expressed a desire for greater planning or predictability of the workload. Variation in the volume of research and variety of topic areas as well as the complexity of decision-making need to be considered when assessing workload. Guideline developers were often clearly passionate about and committed to “*making a difference”* and balanced this with the effort and energy they devoted. 83% of guideline developers rated their experience as ‘Good’ or ‘Excellent’, and cited secondary benefits, such as enhancing their own knowledge.

Where interviewees commented on the future, there was enthusiasm for ensuring the continuation and embedding of the living guidelines approach. However, concerns about sustainability and ongoing funding were raised.

*“How continual is continual for living guidelines?”* (*Guideline developer; Interview Participant 4*).

#### Guideline development and publication technologies and platforms

There were mixed views concerning the technology used to develop and publish the LSGs. Seventy-one per-cent of developers (*n* = 34/44) agreed that ‘The technology supporting production works well’. However, issues with the publishing platform were a source of frustration for some. In general, the benefits of the technology, especially in reducing workload, were recognised.

Most guideline users found it easy to use the LSGs online and liked the way the information was organised. Being able to access the LSGs without a password was thought to be an advantage. Fostering optimal communication with the clinical end-users about the LSGs was viewed as important for dissemination and uptake, especially when updates are made and can be ‘advertised’ to clinicians, health consumers, and policy makers.

*“I like the way they have the strong recommendation, weak recommendation; how they’ve outlined the consensus statements. So, they have put a lot of thought into the way they have structured it and you know, there is a good group of experts that are reviewing all the literature and you know the evidence that’s changing.”* (*Guideline user, Interview Participant 1*).

Guideline developers and users both suggested potential improvements. Developers suggested using an online shared workspace for their reviews rather than email, and exploring ways to boost the current usability, functionality, and integration of MAGICapp with other software. Specific suggestions included developing more sophisticated platforms for reviewing the evidence, streamlining the data editing on the MAGICapp platform, and combining different components to make a single product.

Opportunities for improvement noted by users included increasing search functionality; enabling sharing of guideline sections; and development of a mobile app.

*“I wonder if there should actually be an app… Rather than a page, because that would then reduce the amount of clicks…I think an app-based option where you then can have a better search function would also be better.”* (*Guideline user, Interview Participant 12*).

#### Barriers and facilitators to the development and use of the LSGs

Guideline developers and users (respectively) described barriers and facilitators, as well as potential improvements, to the development and use of LSGs (Table 3).

**Table 3:** Themed barriers and facilitators to the development and use of LSGs

Development		Use	
**Barriers**	**Facilitators**	**Barriers**	**Facilitators**
• High and unpredictable workload • Voluntary contribution **–** threatens sustainability • Lack of cross-talk and integration between different software platforms and collaborative tools	• Collaborative and sustained input from diverse stakeholders • Acknowledgement of contribution • Technical support (e-platforms streamline process)	• Lack of transparency around development group qualifications and potential COIs, decision-making around recommendation inclusion • Potential bias/lack of consensus between agencies • Lack of local context • Usability: o Limited search functionality o Difficulty sharing sections	• Email updates • Online platform • Discipline-specific accessible summaries • Limited acronyms / jargon • Ability to drill down to further evidence • Financial investment • Demonstrated impact (e.g., increased trust, greater use in practice)

## Discussion

The Stroke Foundation’s LSGs were developed in accordance with robust systematic review methods [6, 15, 25, 26], and mirror emerging efforts to implement living guidelines for a range of topic areas [14, 27]. The Stroke Foundation’s living approach to guideline development enabled continual updating of recommendations in line with new evidence, and was considered feasible and acceptable to both developers and users, even for a large complex guideline. This approach was perceived to bring additional value beyond traditional guideline development methods, increasing trust in and credibility of guidelines, and potentially increased guideline use.

The approach employed for LSGs had many strengths, including direct and diverse clinician and stroke survivor/carer involvement; excellent coordination by the team at the Stroke Foundation; and an established core set of development processes and supporting technologies. To the best of our knowledge, this is the first study to evaluate living approaches to guideline development, therefore comparisons with other evaluations is not possible.

Given the resources required to undertake and maintain living guidelines [28], conditions with a high burden of disease and rapidly changing evidence should be prioritised [29]. The work should also be guided by organisations that have well-established relationships with clinicians and people with lived experience [30, 31]. Effective communication strategies and provision of resources to support living guideline adoption as recommendations change need to be embedded in the design early to support evidence-based care [29]. 

Guideline developers identified a need for technological improvements to enable a shared, cohesive work environment and highlighted that the LSGs were developed using tools, including Covidence, and MAGICapp [20, 32], which were originally developed to support traditional guideline development and systematic reviews. Such tools are being rapidly refined to support living guidelines [1, 33]. Developers in our study highlighted the need for technologies to recognise that when updating the living guideline, the unit of publication is the recommendation rather than the entire guideline document, to enable rapid sharing of relevant updates at the level of the recommendation.

Many developers, although not all, felt that the living guideline model had increased their workload, which could impact feasibility of the living guideline model. As more experience is gained through the development and evaluation of this and other living guideline projects, teams may be better able to predict and plan workload to support sustainability. Workloads could be reduced by sharing the work across a larger group; improving efficiency or accuracy of electronic automation tools to free up people to focus on tasks requiring high-level expertise; [33, 34] or harnessing artificial intelligence technology to model guidelines in computer-interpretable and executable formats [35, 36]. End-user uptake of guideline recommendations could be hastened by direct translation into clinical decision support systems and electronic health records as individual implementable items [37, 38]. 

The optimal frequency with which living guidelines and recommendations need to be updated is unknown. Decisions about update frequency are likely to vary among topics based on their clinical importance and how rapidly their evidence-based is changing [39], however, resourcing implications must also be considered [39, 40]. Participants in our study identified facilitators and barriers for living guidelines development and use, and offered insights into how these may be leveraged or overcome to help inform and improve future processes.

### Limitations

Careful interpretation of our Google Analytics data is required as we have no comparative user access data for when new versions of non-living stroke guidelines have been released, and guideline access may not translate to referencing or clinical implementation. The small sample of guideline developers and users working in the context of Australian healthcare delivery systems and the specialised nature of stroke management may limit generalisability of findings. Self-selection bias may have also influenced our results. Our convenience sample may not reflect the perceptions or behaviours of the broader population of clinicians, and especially those who do not routinely use guidelines. The response rate was 34% for the guideline developers which is in-line with previously reported response rates for surveys [41]; however, it is possible that this study appealed to those with strong and/or positive experiences of the development process. Allied health were the largest professional group interviews within the guideline developers, however there was a predominance of speech pathologists, and no occupational therapists. In addition, no nursing representation was captured. Of the developers who were stroke survivors or carers, all had at least six years’ experience meaning there was a lack of representation of people with acute or recent lived experience of stroke. The response rate for guideline users was consistent with expectations for a large-scale email circulation approach [42], but may also have engaged participants with baseline knowledge, perceptions and interest in guidelines. Additional demographic details, such as survey participants’ professional background (guideline developer and user groups) and stroke type and disability (guideline users with lived experience), may have enhanced the completeness and representativeness of our results. Nevertheless, the fact that our survey findings are largely congruent with other related published evidence [29, 39] suggests that respondents are likely to be representative of clinician guideline users [41, 43]. Future evaluation studies should consider other recruitment strategies, such as surveying clinical staff in specialist stroke units in major hospitals. Additional feasibility outcomes should also be adopted, such as those situated within domains of the RE-AIM (Reach, Effectiveness, Adoption, Implementation, Maintenance) framework (e.g., clinical practice indicators, cost measures of implementation and maintenance) [44]. 

## Conclusion

The results of this evaluation demonstrate the feasibility and acceptability of living approaches to guideline development among our sample. Users in our study felt that the LSGs increased trust, access, and use. Evaluations of living guidelines should be undertaken along-side development and implementation to better understand factors that help or hinder development, implementation, adoption, and sustainability.

### Electronic supplementary material

Below is the link to the electronic supplementary material.

Supplementary Material 1

Supplementary Material 2

Supplementary Material 3

Supplementary Material 4

Supplementary Material 5

## Data Availability

The data are thematic coded data derived from qualitative methods, including interviews. Data cannot be shared publicly because they contain potentially personally identifying information. Requests for data may be sent to the Human Research Ethics Committee of Macquarie University, Ground Floor, 16 Wally’s Walk, Sydney, New South Wales, Australia 2109; Email: ethics.secretariat@mq.edu.au; Telephone: +61 2 9850 4194, for researchers who meet the criteria for access to confidential data.
