# Supplementary material for: Is it possible to make ‘living’ guidelines? An evaluation of the Australian Living Stroke Guidelines
Source: BMC Health Serv Res. 2024 Apr 3;24:419. doi: 10.1186/s12913-024-10795-6 (PMC10988967; doi:10.1186/s12913-024-10795-6)
Supplement: Supplementary file 1 — Supplementary Material 1 [file 12913_2024_10795_MOESM1_ESM.docx]

# Additional File 1. Survey tool 1 – Guideline developers

1. Having read the information above, are you willing to participate?
   - Yes (1)
   - No (2) – exit if No

***Background***

1. What is your role in producing living guidelines? (*select as many as apply*))
   - Guideline content development group (1)
   - Consumer expert panel (2)
   - Guideline advisory committee (3)
   - Project team (4)
   - Other (5)
2. How many years have you been involved with guideline production?
3. What is/are your main area/s of interest in the living guidelines process?

***What works well***

1. We are interested in your experience of what works well, what could be improved and what we should do differently in producing the Stroke living guidelines. We are particularly interested how people, process and technology work separately and interact. Please select a response to reflect whether and how strongly you agree/disagree with the following statements:

| **What works well?** | **Strongly agree** | **Agree** | **Neutral** | **Disagree** | **Strongly disagree** |
| --- | --- | --- | --- | --- | --- |
| The way different groups are involved works well |  |  |  |  |  |
| The technology supporting the production process works well |  |  |  |  |  |
| The processes (workflows, communication, etc) underpinning living guideline production work well |  |  |  |  |  |

1. Thinking about how **people** are involved in producing the LSGs…
   1. What works well?
   2. What could be improved?
2. Thinking about how **technology** supports production of the LSGs…
   1. What works well?
   2. What could be improved?
3. Thinking about how processes (workflows, communication, etc.) support production of the LSGs…
   1. What works well?
   2. What could be improved?
4. How has the living guideline process impacted on your workload compared to traditional guideline development?
   - Substantial increase
   - Small increase
   - No change
   - Small decrease
   - Substantial decrease
   - Unsure
5. Please comment. If increased or decreased, why has this change occurred?
6. How would you rate your living guideline development experience?

(e.g. your overall enjoyment of the process, depth of discussions and decision-making, ease of reaching consensus, etc.)

- - Excellent
  - Good
  - Fair
  - Poor
  - Very poor
  - Opportunities to improve

1. What do you see as the biggest opportunities to improve the current living guideline production process?
2. What else should we consider when evaluating the living guidelines production process?
3. What else should we consider when disseminating living guidelines?
4. Can we contact you to find out more about your experiences in living guideline production?
   - Yes
   - No
5. *Answer if ‘Yes’ is selected as a response to Q15. “Can we contact you to find out more about your experiences in living guideline production?”*

“Thank you” *[free-text response fields below]*

- - Name:
  - Email:
  - Other contact information:

Thank you very much for your time and expertise - we really appreciate it.

Please feel free to contact us if you would like to share any further information. [insert contact info]
