# Supplementary material for: Is it possible to make ‘living’ guidelines? An evaluation of the Australian Living Stroke Guidelines
Source: BMC Health Serv Res. 2024 Apr 3;24:419. doi: 10.1186/s12913-024-10795-6 (PMC10988967; doi:10.1186/s12913-024-10795-6)
Supplement: Supplementary file 2 — Supplementary Material 2 [file 12913_2024_10795_MOESM2_ESM.docx]

# Additional File 2. Survey 2 tool – Guideline users

1. Q1. Having read the Participant Information above, are you willing to participate?

- Yes
- No – exit

1. ***Participant demographics*:**

- Age:
- Years of professional experience:
- Years of stroke specific experience:

1. ***Background***
2. Are you aware of the Stroke Living Guidelines? (select as many as apply)

- Yes
- Aware of them but have not used them (go to Q6)
- Not aware of them – exit
- Other - describe

1. What are your reasons for accessing the Stroke Living Guidelines? (select as many as apply)

- Informing my clinical practice for a specific situation/patient
- Informing my clinical practice in general/professional development
- Guiding clinical practice improvement and/or professional development within my team
- As part of guidelines development team
- Inform a literature review for my research/work
- Inform development of a research question/proposal
- Inform development of a research question/ proposal
- Other - describe

1. Have you used previous (static) versions of the Stroke guidelines?

- Used them regularly
- Used them occasionally
- Aware of them but did not use them
- Not aware of them
- Other - describe

1. In what clinical contexts do you use Stroke guidelines?

- Acute medical care (including pre-hospital, ED, and other acute care)
- Acute care nursing
- Acute rehabilitation (allied health)
- Rehabilitation medical care
- Rehabilitation nursing
- Rehabilitation therapy (allied health)
- Other - describe

1. How do you usually access the Stroke guidelines?

- PC/Laptop
- Tablet
- Mobile Phone
- Other - describe

***Using the guidelines***

Stroke guidelines have recently moved to ‘living’ mode. Living guidelines are guidelines that are continually updated as new evidence become available. In the past the stroke guidelines were updated in 2007, 2010 and 2017.

1. How has the introduction of the living guideline process impacted on your trust in the guidelines compared to traditional guideline development?
   - Substantial increase
   - Small increase
   - No change
   - Small decrease
   - Substantial decrease
   1. Why?
2. How has the living guideline process impacted on how likely you are to access the guidelines compared to traditional (static) guidelines?

- Substantial increase
- Small increase
- No change
- Small decrease
- Substantial decrease
  1. Why?

1. How has the living guideline process impacted on the frequency with which you will access the guidelines compared to traditional (static) guidelines?

- Substantial increase
- Small increase
- No change
- Small decrease
- Substantial decrease
  1. Why?

1. How has the living guideline process impacted on how likely you are to follow the guideline recommendations compared to traditional (static) guidelines?

- Substantial increase
- Small increase
- No change
- Small decrease
- Substantial decrease
  1. Why?

1. Do the current Stroke living guidelines address relevant clinical topics?

- Yes, all the relevant questions are included
- Yes, most of the important questions, and few irrelevant questions are included
- Mixed, some of the important questions, and some irrelevant questions are included
- No, few of the important questions, and mostly irrelevant questions are included
- No, none of the relevant questions are included

1. How could the topics covered by the guidelines be improved? What areas are missing? Which should be removed? *[free text response field]*

***Thank you for participating*** *[end survey]*
