# Supplementary material for: Is it possible to make ‘living’ guidelines? An evaluation of the Australian Living Stroke Guidelines
Source: BMC Health Serv Res. 2024 Apr 3;24:419. doi: 10.1186/s12913-024-10795-6 (PMC10988967; doi:10.1186/s12913-024-10795-6)
Supplement: Supplementary file 4 — Supplementary Material 4 [file 12913_2024_10795_MOESM4_ESM.docx]

# Additional File 4. Interview schedule 1 – Guideline developers

Thank you for agreeing to talk with us – we really appreciate your time and insights.

This research explores the benefits, challenges and opportunities of producing living, continually updated, guidelines. This information will be used to improve approaches to producing living guidelines for further piloting and scale-up.

I’d like to start by understanding a little about your background:

1. Can you tell me about your experience with evidence synthesis and guideline production?
   (If not provided, prompt for duration of experience, types of roles, etc)
2. What experience with guideline production did you have before your involvement in LSGs?
   (Prompt for context of conduct of described review models including funding, setting, NHMRC endorsement, etc)

*We are particularly interested in your experience of contributing to the Stroke living guidelines work:*

1. *What has been your role in this process?*(*If not provided, prompt for start date, duration of involvement, types of tasks, etc*)

***Producing living guidelines***

1. How well do you think the team is currently doing in producing living guidelines? [Prompt: Consider using a 1-10 scale]
2. In your experience, what has worked well in producing the stroke living guidelines? [Prompt: Why?]
3. What was most helpful in transitioning to/updating living guidelines?
4. What parts of the process could be improved? [Prompt: Why? How?]
5. As the work continues, what do you think should be done differently? [Prompt: How?]
6. What have been the biggest challenges so far? [Prompt: Internal? External?]
7. What have been the biggest benefits so far?
8. What impact has the living guideline approach had on your role? [Prompt: Types of work? Workload? Changes over time?]
9. How would you describe the quality of your experience? [Prompt: Consider using a 1-10 scale]

***We are looking to find opportunities to improve how living guidelines are produced and translated into practice.***

1. *What are the biggest opportunities to improve living guidelines? [Prompt: Production* processes? Dissemination? Uptake in practice? End-user experience? What strategies were used/how was this done? How could it be improved?]
2. What else should we consider when exploring the concept of and/or evaluating living guidelines?
3. Do you have suggestions of others we should speak to about living guidelines?

***Close & thanks.***
