# Supplementary material for: Is it possible to make ‘living’ guidelines? An evaluation of the Australian Living Stroke Guidelines
Source: BMC Health Serv Res. 2024 Apr 3;24:419. doi: 10.1186/s12913-024-10795-6 (PMC10988967; doi:10.1186/s12913-024-10795-6)
Supplement: Supplementary file 5 — Supplementary Material 5 [file 12913_2024_10795_MOESM5_ESM.docx]

# Additional File 5. Interview schedule 2 – Guideline users.

1. I’d like to start by understanding a little about your background
   1. Can you please describe your clinical role?
   2. Have you had any experience with evidence-based practice, evidence synthesis or guideline production?
      (*If not provided, prompt for duration of experience, types of roles, etc*)
   3. Have you led or participated in QI activities that refer to stroke guidelines?
   4. How do you use guidelines in your work?
2. We are interested in your reflections on the idea of living guidelines, which are continually updated to reflect new evidence:
   1. What has been your understanding of the term “living guidelines”?
   2. What appeals to you about the idea of living guidelines?
   3. What concerns you about the idea of living guidelines?
   4. Do you prefer living guidelines, or periodically updated static guidelines?

[*Prompt: Why?*]

1. I am going to ask you a few questions, first about your experience and reflections on the Stroke guidelines in general, and then focusing on how they are shared and translated into practice.
   1. Thinking about the Stroke living guidelines in general:
      1. How did you learn about the guidelines? *[exit point if they haven’t heard about them]*
      2. How have you used the guidelines? *[exit point if they haven’t used them]*
      3. What do you like about the LSGs?
      4. What could be improved?

*[Consider a range of possible prompts here to identify different parts of the process where things can go wrong? Ensuring a broad range of experts* (*including consumers*) *are involved and being transparent about possible conflicts of interest; Finding and summarising latest evidence; Getting wider views of guideline changes during public consultation; Improved ways of communicating changes; Improved presentation of information on website including making it easier to access the information]*

- - 1. As the work continues, what do you think should be done differently?
    2. What do you think are the biggest opportunities to improve production and/or usability of living guidelines?
  1. Thinking about how the Stroke living guidelines are shared and translated into practice in particular:
     1. What is working well in how the living guidelines are shared and translated into practice? [*Prompts: How can we promote use of the Living Guideline by clinicians to guide their everyday practice? How can we promote use of the Living Guideline by researchers?*]
     2. What could be improved?
     3. What have been the biggest benefits so far in using the living guidelines?
     4. What have been the biggest challenges so far in using the living guidelines?
     5. How does the living approach effect the credibility/acceptability of the Stroke guidelines?
     6. What are the biggest opportunities to improve communication and implementation of living guidelines?

1. We are looking for opportunities to improve how living guidelines are produced and shared.
   1. What else should we consider when exploring the concept of living guidelines during this evaluation?
   2. Do you have suggestions of others we should speak to about living guidelines?

***Close & thanks.***
